# Systematic review and meta‐analysis of the effect of exercise training on asprosin in randomized controlled trials

**DOI:** 10.14814/phy2.70392

**Published:** 2025-06-13

**Authors:** Mohammad Rahman Rahimi, Michael E. Symonds, Hassan Faraji, Hadi Golpasandi

**Affiliations:** ^1^ Department of Exercise Physiology University of Kurdistan Sanandaj Iran; ^2^ Academic Unit of Population and Lifespan Sciences, Centre for Perinatal Research School of Medicine, University of Nottingham Nottingham UK; ^3^ Department of Physical Education and Sports Science Marivan Branch, Islamic Azad University Marivan Iran

**Keywords:** asprosin, exercise training, high‐intensity interval training, obesity

## Abstract

Asprosin, a protein that enhances insulin resistance by stimulating hepatic glucose secretion, is linked to obesity, metabolic disorders, and cardiovascular diseases. This systematic review and meta‐analysis investigate the influence of exercise training (ET) on circulating concentrations of asprosin. A systematic search of PubMed, Web of Science, and Google Scholar databases was conducted for all randomized controlled clinical trials from January 2016 to November 2024. A total of 431 relevant articles were retrieved and screened according to the study population, intervention method, and study type, resulting in the selection of 14 articles for the meta‐analysis. All statistical results were analyzed using Comprehensive Meta‐Analysis Software version 2 (CMA2). The overall effect size, using Hedges' g, based on the random effects model for asprosin with ET, was −1.70 (95% CI −2.17 to −1.23, *p* = 0.0001). A decrease in asprosin concentration was observed with all types of ET including aerobic training (AT, *H* = −1.71, *p* = 0.0001), high‐intensity interval training (HIIT, *H* = −1.81, *p* = 0.001), and resistance training (RT, *H* = −1.62, *p* = 0.0001). Furthermore, subgroup analysis showed differences in asprosin based on body mass index (*Q*‐value = 11.09, df = 2, *p* = 0.004) and health status of the subjects (*Q*‐value = 12.64, df = 2, *p* = 0.002); however, there were no differences based on sex (*p* = 0.67), types (*p* = 0.96), and duration (*p* = 0.34) of training. Our findings suggest that ET is associated with a decrease in circulating asprosin that could have a beneficial effect in preventing metabolic disease that is enhanced with obesity. Systematic review registration PROSPERO: CRD42023468813.

## 1 INTRODUCTION

Obesity is associated with numerous health conditions, including cardiovascular disease, diabetes, and metabolic syndrome (Organization, W. H, 2021). Adipose tissue, the body's largest endocrine tissue, plays a significant role in appetite regulation by secreting numerous obesity‐related adipokines such as leptin, adiponectin, and resistin (Huang et al., 2022). In 2016, a 140‐amino acid adipokine named asprosin was discovered, also known as lenomorelin or the “hunger hormone,” it acts as a central appetite stimulator (Liu et al., 2022). Asprosin acts on the liver, inducing gluconeogenesis via the G‐protein coupled receptors (GPCR)‐activated cAMP signaling pathway, by activating the PKA‐cAMP‐G protein pathway through the olfactory receptor (OLFR7342) (Li et al., 2019). Asprosin can also cross the blood–brain barrier and influence the central nervous system (CNS), thereby activating appetite‐stimulating AgRP neurons in the hypothalamus's arcuate nucleus while simultaneously inhibiting the appetite‐suppressing POMC neurons, thereby modulating appetite (Duerrschmid et al., 2017). With overweight and obesity, asprosin is 2–4 times higher than in normal weight adults and increases with hunger and low blood glucose (Ugur & Aydin, 2019). Both type 2 diabetes and impaired glucose regulation are associated with raised asprosin, together with impaired insulin sensitivity and lipid metabolism (Wiecek et al., 2018).

Limited studies have explored the effect of chronic exercise in humans on asprosin, with most focusing on animal models, for example, 8 weeks of aerobic training in diabetic rats reducing hepatic asprosin (Ko et al., 2019), as does 2 months of swimming training (Nakhaei et al., 2019). In adult humans, 8 weeks of high‐intensity circular resistance training (RT) reduced asprosin in overweight and obese women (Dolataabadi et al., 2023a), as does intermittent RT in conjunction with improved body composition (Jahangiri et al., 2021) but the extent to which such responses may be modulated by exercise type and body composition is less clear.

Furthermore, asprosin, a glucogenic adipokine derived from fibrillin‐1 (FBN1), promotes hepatic glucose release and is elevated in metabolic disorders (e.g., obesity, insulin resistance). Its reduction could improve metabolic health by counteracting hyperglycemia and inflammation. Exercise training may lower asprosin by: (1) reducing visceral fat (its main source; Romere et al., 2016), (2) inducing anti‐inflammatory myokines (IL‐6/IL‐10) that suppress its expression (Mazur‐Bialy, 2023; Yuan et al., 2020), and (3) enhancing insulin signaling to inhibit its glucogenic actions via AMPK/PGC‐1α (Ko et al., 2019; Liu et al., 2022). We therefore undertook a systematic review and meta‐analysis to further examine the effects of exercise training (ET) on asprosin.

## 2 METHODS

### 2.1 Eligibility criteria

Our meta‐analysis protocol was developed based on the Preferred Reporting Items for Systematic Reviews and Meta‐Analyses (PRISMA) guidelines. The protocol was also verified on the PROSPERO website to ensure uniqueness and was registered under the code CRD42023468813. This article presented a review and meta‐analysis using the PICO (Participant‐Intervention‐Comparator‐Outcomes) framework guidelines. The inclusion criteria are (a) Population: studies involving human subjects aged 18 years and above, irrespective of sex and health status, (b) Intervention: studies demonstrating the effect of ET with a duration of exercise intervention equal to or more than 4 weeks, (c) Comparison: Randomized Controlled Trials (RCTs) with a control group, (d) Outcomes: studies where asprosin was measured.

### 2.2 Publication search strategy

In this meta‐analysis, the Boolean Logic method was employed to select eligible articles, specifically using the connectors “And,” “Or,” and “Not,” in a systematic search across PubMed, Web of Science, and Google Scholar databases on publications in the English language. The search began from 2016 using the keywords “Sports exercises” and “Asprosin”. Keywords used for exercise training included: “Exercise”, “Exercise training”, “Training”, “Physical activity”, “High intensity interval training [HIIT]”, “Resistance training”, “Weight training”, “Aerobic training”, and “Anaerobic training”. The search covered all studies from 2016 to 2024, given that asprosin was discovered in 2016. The keywords for asprosin included “Asprosin” and “the C‐terminal cleavage product of profibrillin”. There were no language restrictions for the search, and eligible articles in any language were included in this meta‐analysis.

### 2.3 Study selection

**FIGURE 1:**
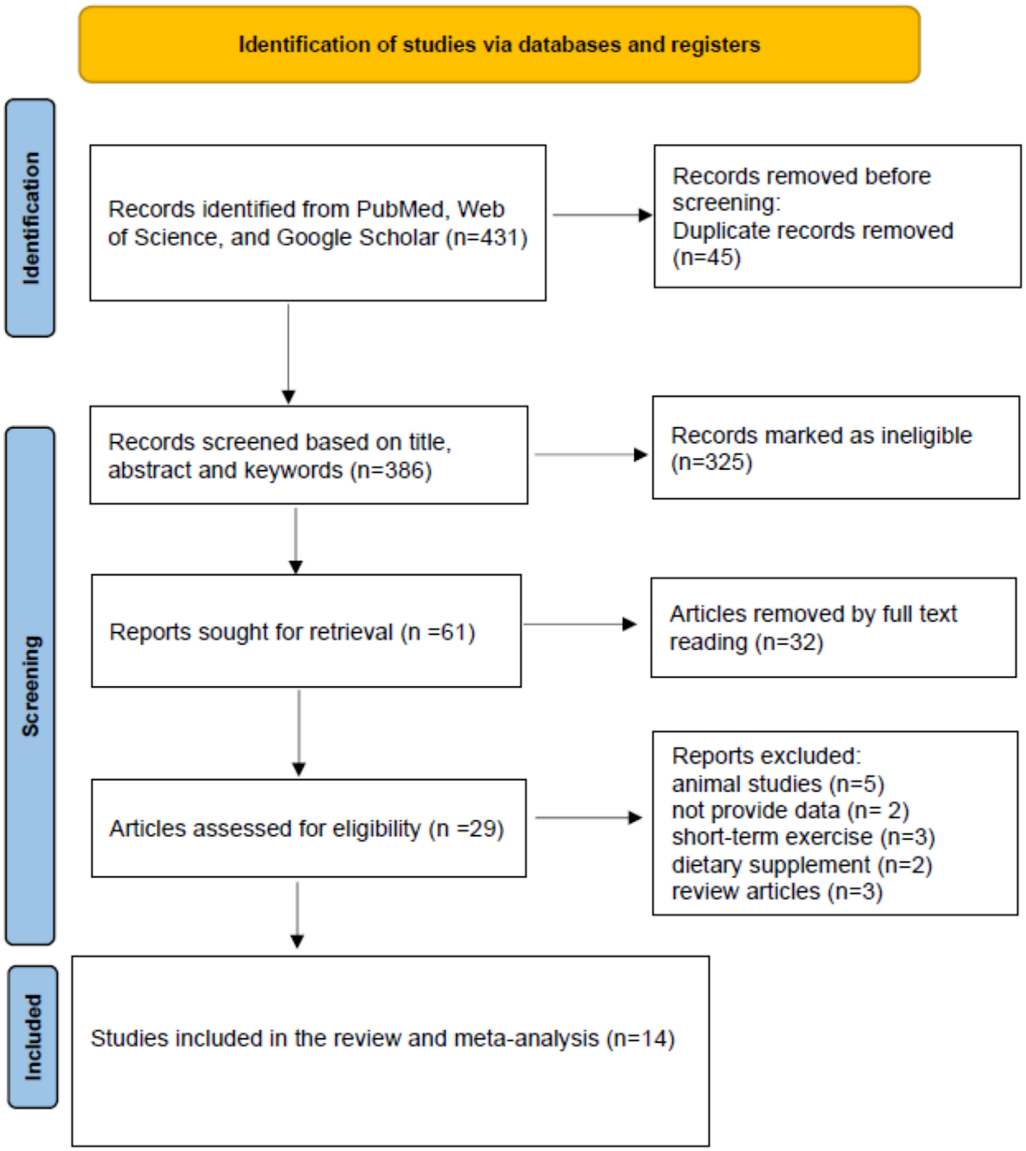
PRISMA flowchart indicating the study selection process for investigating the effect of exercise training on asprosin (Page et al., 2021).

All English language articles identified in the search were imported into EndNote software version 20. Initially, duplicate articles were removed. Subsequent screening was conducted using the title, abstract, and keywords. The remaining articles underwent a secondary screening by reviewing the full text to determine if they met the criteria for inclusion, that is, studies that included obese individuals with or without chronic diseases and healthy individuals were included. Regarding ET, there were no restrictions on the type or intensity of training, but the duration of the training protocol was not less than 4 weeks, and RCTs were included. Exclusion criteria included: animal studies, studies with an exercise intervention duration of less than 4 weeks, review articles, conference presentations, and studies that included ET as part of a multicomponent treatment (e.g., ET with dietary intervention). All steps of study selection were independently performed by two authors (M.R.R. and H.F.), and any disagreements were resolved through consultation with a colleague at our university (H.G.). The systematic and comprehensive search process of studies, along with the inclusion or exclusion criteria of studies, is specified in the PRISMA 2020 flow chart (Figure 1, Appendix S1).

### 2.4 Data extraction process

Descriptive information and data required for meta‐analysis were extracted from all the final studies eligible for inclusion in the current research (Table 1). This information includes: (a) study type, (b) publication year and first author, (c) subject characteristics including age, body mass index (BMI), and health status, (d) ET characteristics including type, duration, and number of sessions per week, (e) mean and standard deviation of asprosin values in the pre‐test and post‐test stages in the training and control groups, and the sample size. If needed, GetData software was used to extract data from graphs (Akbulut et al., 2022; Ghalavand et al., 2023; Jahangiri et al., 2021; Kantorowicz et al., 2021). The data extraction process was jointly performed by two authors (M.R.R. and H.F.), and any disagreements were resolved through consultation with another author (B.L.).

**TABLE 1:** Characteristics of the intervention used within each study included in the meta‐analysis.

Number	Study, year, country	Study design	Subjects/sex	Sample size (training/control)	Age (mean: Years)	BMI (kg/m^2^)	Type of exercise training	Sessions per week/Total training duration	Reduced Asprosin
1	Akbulut, 2022, Turkey (Akbulut et al., 2022)	RCT	HL/M	AT:30, HIIT:30, RT:30/CO:30	AT:20.43, HIIT:21.19, RT:20.93/CO: 20.60	AT:22.50, HIIT:23.87, RT:23.47/CO: 23.67	AT, HIIT, RT	3 sessions/8 weeks	Yes
2	Dolataabadi, 2022, Iran (Dolataabadi et al., 2023b)	RCT	OWOB/F	HIIT: 15/CO:15	HIIT/CO:30–45	HIIT:32.32/CO:32.7	HIIT	3 sessions/8 weeks	Yes
3	Dolataabadi, 2023, Iran (Dolataabadi et al., 2023a)	RCT	OWOB/F	HICRT:15/CO:15	HICRT/CO:30–45	HICRT:31.18/CO:32.72	HICRT	3 sessions/8 weeks	Yes
4	Ghalavand, 2023, Iran (Ghalavand et al., 2023)	RCT	DIB/M	AT:10/CO:10	AT:39.61/CO:38.12	AT:26.99/CO:27.39	AT	3 sessions/12 weeks	Yes
5	Ghalavand, 2024, Iran (Ghalavand et al., 2024)	RCT	DIB/F	AT:15/CO:15	AT:26.8/CO:39.1	AT:26.8/CO:26.6	AT	3 sessions/3 weeks	Yes
6	Jahangiri, 2021, Iran (Jahangiri et al., 2021)	RCT	OWOB/M	TRT:11, CRT:11, IRT:11/CO:11	TRT:25.71, CRT:26.31, IRT:26.54/CO:26.54	TRT:32.48, CRT:33.03, IRT:33.14/CO:32.90	TRT, CRT, IRT	3 sessions/12 weeks	Yes
7	Kantorowicz, 2021, Poland (Kantorowicz et al., 2021)	RCT	OWOB/F	AT, CO:14	AT/CO:30.14	AT/CO: 33.85	AT (NW)	3 sessions/8 weeks	Yes
8	Hemmati Moghadam, 2023, Iran (Hemati Moghadam et al., 2023)	RCT	OWOB/M	HIIT:12/CO:12	HIIT: 43.92/CO:44.58	HIIT: 32.93/CO:31.60	HIIT	3 sessions/6 weeks	Yes
9	Nakhaei, 2023, Iran (Nakhaei et al., 2023)	RCT	OWOB/F	ATSP:15, ATSB:15/CO:15	ATSP:37.20, ATSB:33.46/CO:35.86	ATSP:29.08, ATSB:30.61/CO:29.17	ATSP, ATSB	3 sessions/6 weeks	Yes
10	Salehi 2023b, Iran (Salehi et al., 2023b)	RCT	OWOB/F	AT:12/CO:12	AT:41.33/CO:41.00	AT:31.46/CO:32.2	AT (MICT)	3 sessions/10 weeks	Yes
11	Salehi 2023, Iran (Salehi et al., 2023a)	RCT	OWOB/F	HIIT:12/CO:12	HIIT:40.97/CO:41.00	NOT REPORTED	HIIT	3 sessions/10 weeks	Yes
12	Suder 2024, Poland. (Suder et al., 2024)	RCT	OWOB/M	AT:21/RT:21/CO:20	AT:34.21/RT:37.37/CO:38.26	AT:34.57/RT:33.14/CO:33.20	AT/RT	3 sessions/12 weeks	Yes
13	Zarei, 2021, Iran (Zarei et al., 2021)	RCT	DIB/M	AT/RT:12/CO:12	AT/RT:48.7/CO:49.8	AT/RT:26.69/CO:27.21	AT/RT	3 sessions/12 weeks	Yes
14	Zarei 2023, Iran (Zarei & Nakhzari Khodakheir, 2023)	RCT	OWOB/M	HIIT:10/RT:11/CO:10	HIIT:43.5/RT:41.5/CO:43.08	HIIT:30.05/RT:29.71/CO:30.94	HIIT/RT	3 sessions/9 weeks	Yes

Abbreviations: AT, aerobic training; ATRT, Combined resistance aerobic exercise; ATSB, stationary bike; ATSP, spinning (cycling with music); CRT, circular resistance training; Co, Control Group; DIB, Diabetics; HIC RT, high intensity circuit resistance training; HIIT, high intensity interval training; IRT, interval resistance training; NW, Nordic walking training; RCT, randomized controlled trial; RT, resistance training; TRT, traditional resistance training.

### 2.5 Quality assessment of studies

**FIGURE 2:**
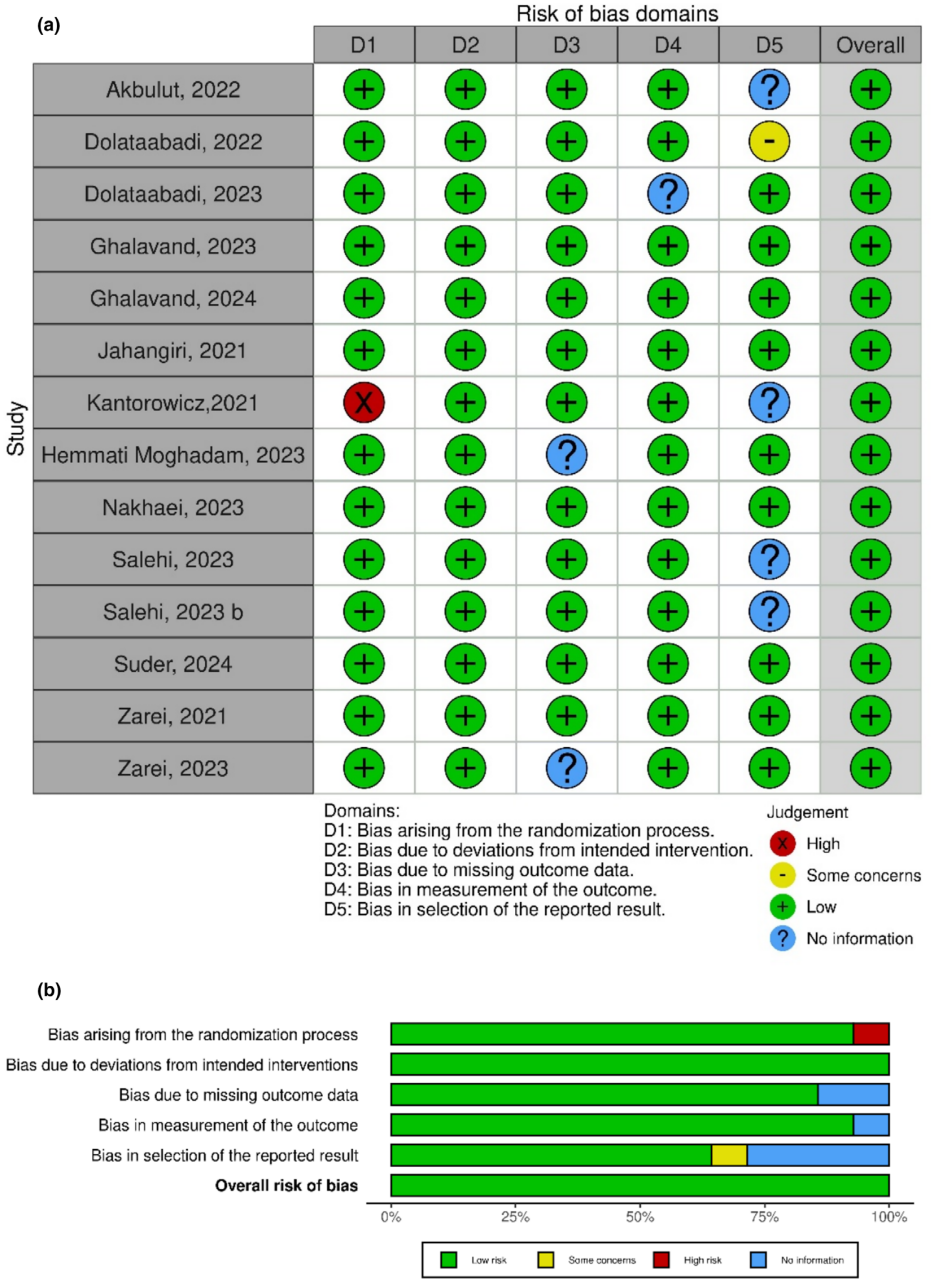
Risk of (a) bias and (b) summary graphs within studies.

In this meta‐analysis, the PEDro checklist was used to assess the quality of the selected studies (Verhagen et al., 1998). This checklist has 11 items, two of which, that is, blinding of the subjects and blinding of the interventionist, were removed due to their non‐implementation in exercise interventions. As such, nine items were used to evaluate the quality of studies. The quality of the studies was independently evaluated by two authors (M.R.R. and H.F.), and any disagreements were resolved through consultation with another author. In case of compliance with the desired criteria, the number one (1) was used, non‐compliance was marked with zero (0), and lack of clarity was marked with a question mark (?). The score provided for the quality of the studies ranged from zero to a maximum of 9, with a higher score indicating a higher quality of the study (Table 2). Also, the Risk of Bias 2 Tool (Cochrane RoB 2) was used to assess the risk of bias and the quality of each study (Figure 2), which includes a table with 5 evaluation areas: (1) randomization process, (2) deviation from the desired intervention, (3) missing outcome data, (4) measurement of the outcome, (5) selection of the reported results (Sterne et al., 2019). Each domain is graded as “low bias”, “some concern”, or “high risk of bias”.

**TABLE 2:** Summary of the method quality assessment as determined by the PEDro scale.

Study	Q1	Q2	Q3	Q4	Q5	Q6	Q7	Q8	Q9	Q10	Q11	Total score
Akbulut, 2022	1	1	?	1	N.APP	N.APP	1	1	1	1	1	8
Dolataabadi, 2022	1	1	1	1	N.APP	N.APP	1	1	1	1	1	9
Dolataabadi, 2023	1	1	?	1	N.APP	N.APP	?	1	1	1	1	7
Ghalavand, 2023	1	1	?	1	N.APP	N.APP	0	1	1	1	1	7
Ghalavand, 2024												
Jahangiri, 2021	1	1	1	1	N.APP	N.APP	1	1	1	1	1	9
Kantorowicz,2021	0	1	?	1	N.APP	N.APP	0	1	1	1	1	6
Hemmati Moghadam, 2023	1	1	1	1	N.APP	N.APP	1	1	1	1	1	9
Nakhaei, 2023	1	1	?	1	N.APP	N.APP	1	1	1	1	1	8
Salehi 2023b	1	1	1	1	N.APP	N.APP	1	1	1	1	1	9
Salehi 2023	1	1	1	1	N.APP	N.APP	1	1	1	1	1	9
Suder 2024	1	1	?	1	N.APP	N.APP	1	1	1	1	1	8
Zarei, 2021	1	1	?	1	N.APP	N.APP	1	1	1	1	1	8
Zarei, 2023	1	1	1	1	N.APP	N.APP	1	1	1	1	1	9

### 2.6 Data synthesis and statistical analysis

Due to the small sample size within all available studies, Hedges' g and 95% confidence intervals (CIs) were used to calculate the effect size. The overall effect size was calculated using Hedges' g by comparing the mean and standard deviation of the training group against controls and the sample size of both groups. The interpretation of Hedges' g values was based on Cochran's guidelines, where an effect size of 0.2 is considered small, 0.5 medium, and 0.8 large. To check the heterogeneity between eligible studies, the I2 test and the significance of the *Q* test were used. I2 values less than 50% indicate low heterogeneity, and more than 50% indicate high heterogeneity. In case of high heterogeneity, the random effects model was used to calculate the overall effect size (Hedges *g* values). Also, to check for publication bias, visual analysis of the Funnel Plot as well as the Egger, Begg, and Mezomdar tests were used. If publication bias was observed by visual analysis of the funnel plot, the trim‐and‐fill method was used to correct it. Also, subgroup analysis was performed based on the type of exercise (aerobic, anaerobic, and resistance), sex (male and female), and duration of exercise (less than 8 weeks versus more than 8 weeks). Statistical analyses were performed by CMA3 software (Borenstein, 2022).

## 3 RESULTS

### 3.1 Search findings

During the database search conducted up until November 10, 2024, we identified 431 studies. After eliminating 45 duplicates, we were left with 386 studies for initial screening. At this stage, we excluded 325 studies based on the examination of their titles and abstracts, leaving us with 61 studies. We then downloaded and reviewed the full text of these 61 studies, which led to the exclusion of 32 more studies, leaving us with 29. Five animal‐based studies were excluded, as were 2 with insufficient data, 3 that focused on short‐term exercise, 2 using a dietary supplement group, and 3 review studies. As a result, we were left with 14 eligible studies, all of which compared an ET and control group. Among the selected studies, 5 had more than one training group (Akbulut et al., 2022; Jahangiri et al., 2021; Nakhaei et al., 2022), and one had two training periods of 4 and 8 weeks (Kantorowicz et al., 2021). Regarding sex, half of the studies investigated the effect of exercise on asprosin in males, while the other half focused on females. The quality scores of the studies included in this research are demonstrated in Table 2.

### 3.2 Meta‐analysis findings

**FIGURE 3:**
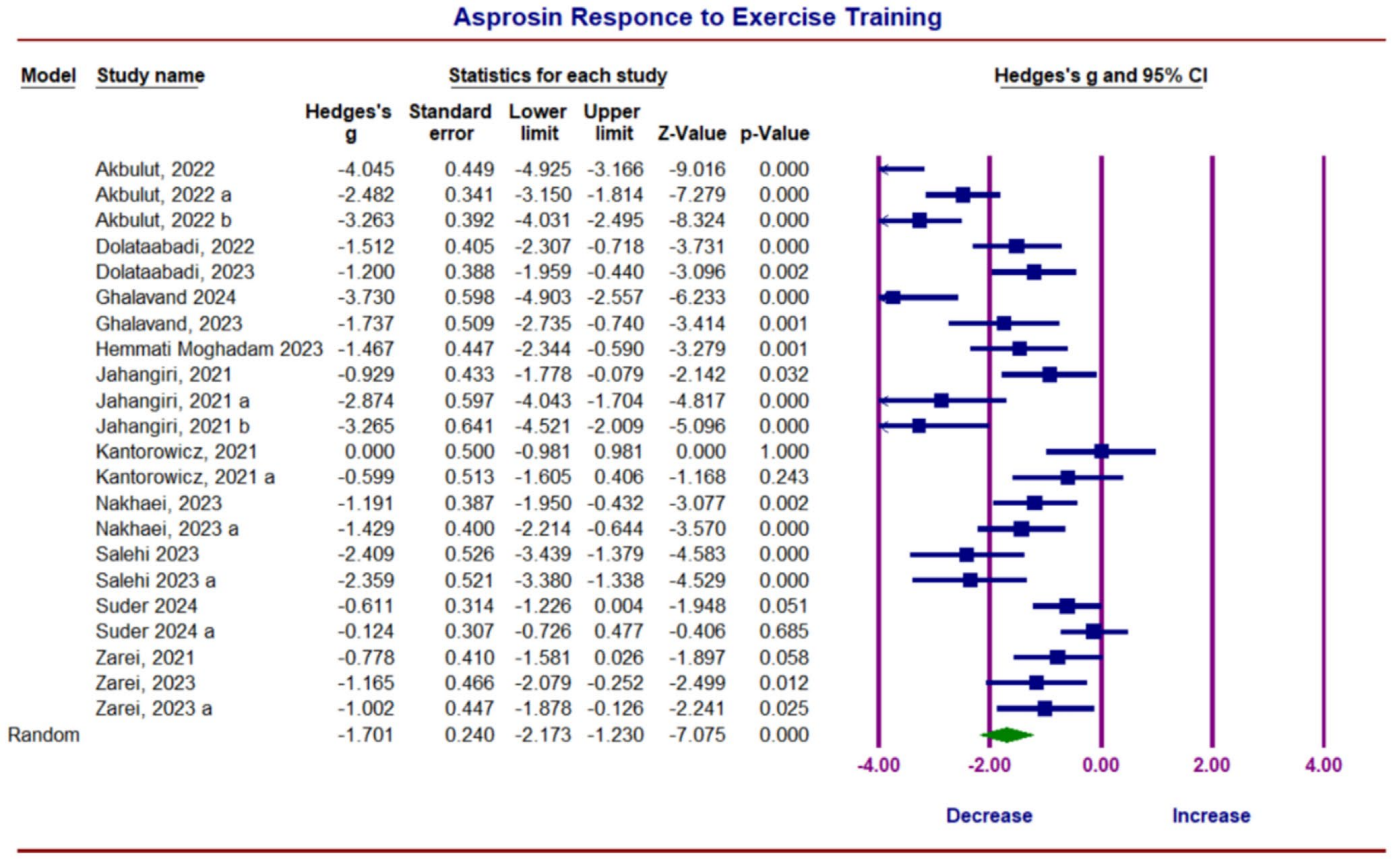
Forest plot represents the summary findings from 14 studies that investigated the effect of exercise training on asprosin in human blood.

**FIGURE 4:**
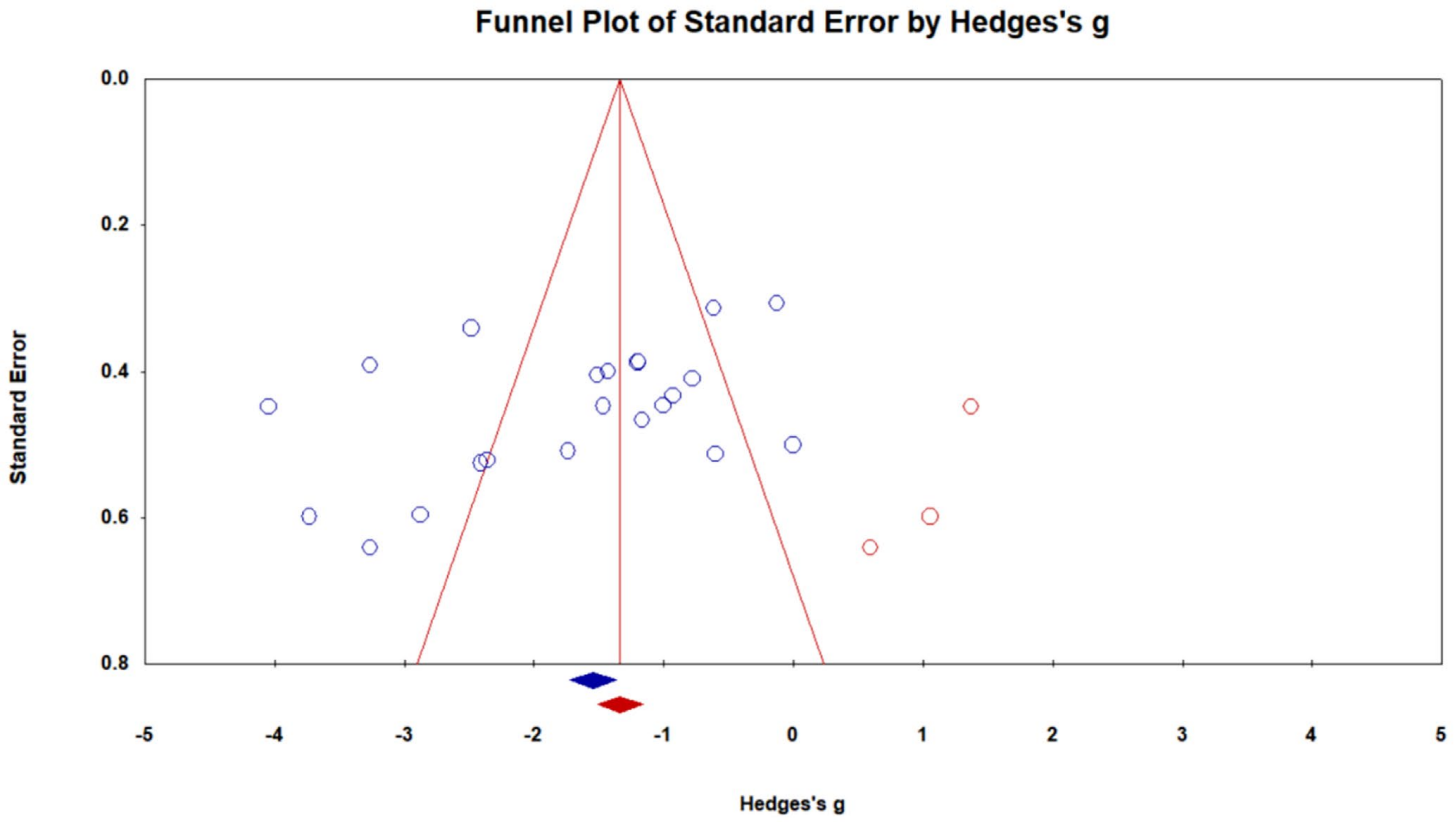
The funnel plot depicts the meta‐analysis findings from 14 studies investigating the effect of exercise training on asprosin concentration. The studies imputed via the Trim and Fill method are prominently marked in red on the left side of the diagram.

The findings from 14 studies that investigated the effect of ET on asprosin concentrations were included in the meta‐analysis, with combined results showing heterogeneity between studies (*I* ^2^ = 85.11, *p* = 0.0001), so the random model method was used to calculate the overall effect size. The overall Hedges' *g* for asprosin was −1.70 (95% CI −2.17 to −1.23, *p* = 0.0001), indicating a significant decrease in asprosin concentration due to ET (Figure 3). Publication bias was checked using visual analysis of the funnel plot (Figure 4), Egger's test, and Begg and Mazumdar Rank Correlation Test. The results of Egger's Test showed the bias, with an intercept (B0) is −5.74973, 95% confidence interval (−11.28247, −0.21698), with *t* = 2.16777, df = 20 and the 2‐tailed *p*‐value is 0.04242. The Begg and Mazumdar Test showed that Kendall's tau b was −0.33766, with a 1‐tailed *p*‐value (recommended) of 0.01392 or a 2‐tailed *p*‐value of 0.02785. So, under the random effects model, the point estimate and 95% confidence interval for the combined studies was −1.70 (−2.17, −1.22). Using Trim and Fill, the imputed point estimate was −1.39907 (−1.91195, −0.88619) (Figure 4).

### 3.3 Subgroup analysis findings

**FIGURE 5:**
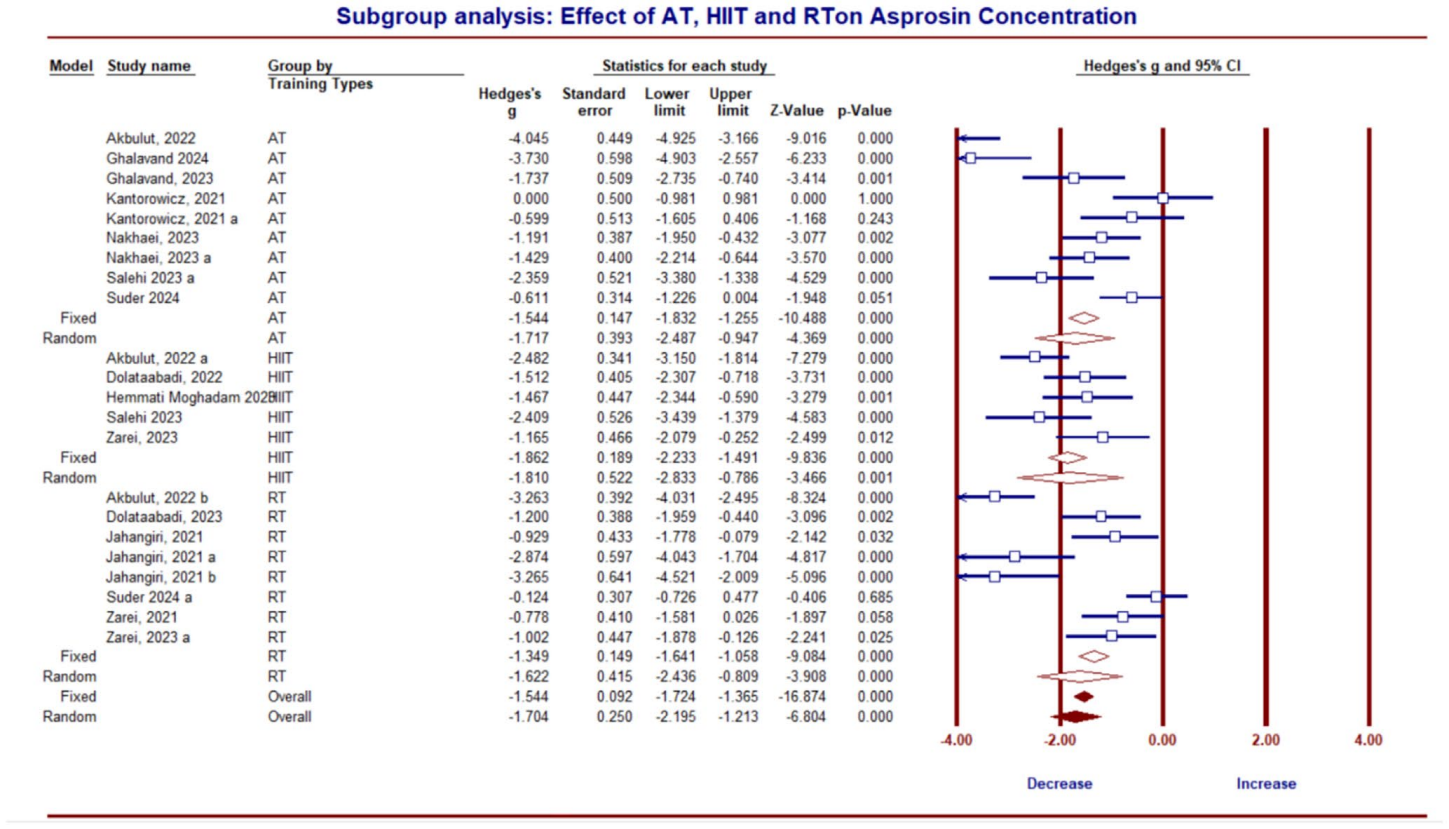
Forest plot illustrating the results of studies examining the impact of exercise training on asprosin in humans, with training types including aerobic training (AT), resistance training (RT), and high‐intensity interval training (HIIT).

The results from the subgroup analysis based on the type of ET revealed no significant difference in the impact of three training methods (AT, anaerobic training, and RT) on asprosin (*Q*‐value = 0.081, df = 2, *p* = 0.96). However, all training methods, including AT (*g* = −1.71; 95% CI, −2.48 to −0.94; *p* = 0.0001; *I* ^2^ = 88.51%), HIIT (*g* = −1.86; 95% CI, −2.23 to −1.49; *p* = 0.0001; *I* ^2^ = 50.86%), and RT (*g* = −1.62; 95% CI, −2.43 to −0.80; *p* = 0.0001; *I* ^2^ = 88.11%), significantly decreased asprosin (Figure 5).

**FIGURE 6:**
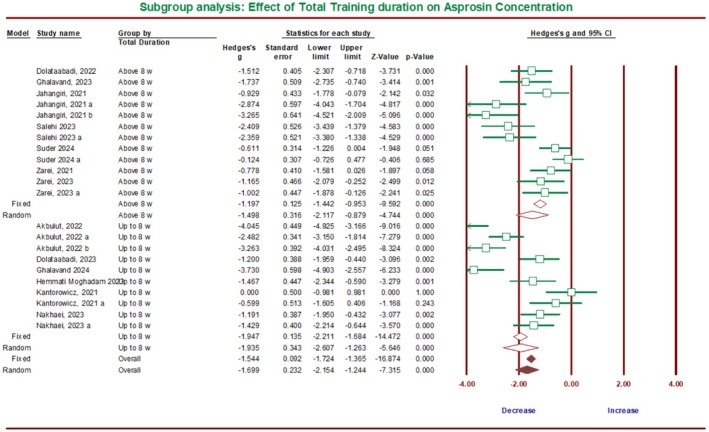
Forest plot summarizing subgroup analysis based on the total duration of training and its effect on asprosin protein in humans.

The subgroup analysis related to the effect of exercise duration on asprosin considered two periods: either less than 8 weeks or equal to or more than 8 weeks (Figure 6). The results showed a significant reduction of asprosin in ET for equal to and less than 8 weeks, with the overall effect size of Hedges' g equal to −1.93 (95% CI −2.60 to −1.26, *p* = 0.0001; *I* ^2^ = 88.29%) and in exercise more than 8 weeks with the overall effect size of Hedges' *g* equal to −1.49 (95% CI −2.11 to −0.87, *p* = 0.0001; *I* ^2^ = 76.93%). No significant difference was observed between these two training periods (*Q*‐value = 0.87, df = 1, *p* = 0.34).

**FIGURE 7:**
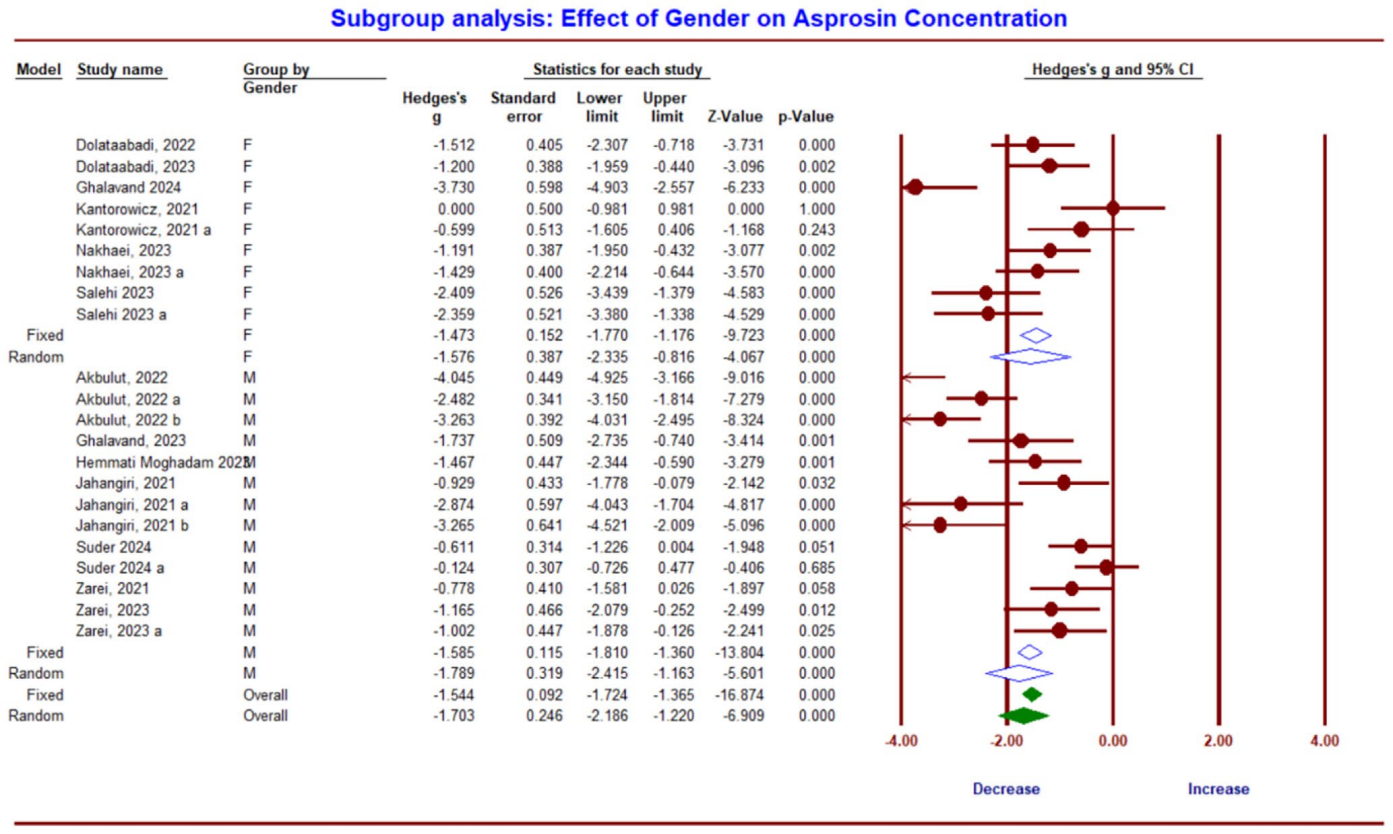
Forest plot summarizing subgroup analysis based on sex and its influence on asprosin protein response to exercise training in humans.

The subgroup analysis based on the sex of the subjects indicates no significant difference in asprosin between sexes due to ET (*Q*‐value = 0.18, df = 1, *p* = 0.67). However, within‐group changes showed a significant decrease in asprosin concentration due to ET in both male (*g* = −1.78; 95% CI, −2.41 to −1.16; *p* = 0.0001; *I* ^2^ = 88.89%) and female (*g* = −1.57; 95% CI, −2.33 to −0.816; *p* = 0.0001; *I* ^2^ = 75.68%) (Figure 7).

**FIGURE 8:**
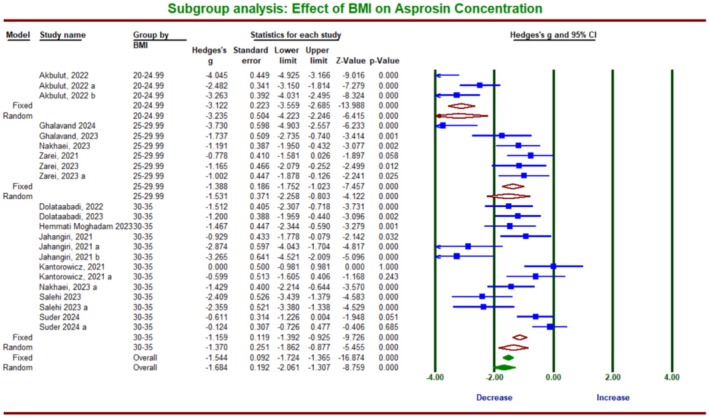
Forest plot summarizing subgroup analysis based on body mass index (BMI) and its impact on asprosin protein due to exercise training in humans.

The subgroup analysis, based on the BMI of the participants, revealed significant differences in asprosin due to ET between 2 BMI categories: BMI = 25–29.95 and BMI = 30–35 (*Q*‐value = 11.198, df = 2, *p* = 0.004) (Figure 8). Only one article had a BMI of 20 to 24.9, which was excluded from the analysis. The within‐group changes demonstrated a significant reduction in asprosin due to ET in BMI = 25–29.95 (*g* = −1.53; 95% CI, −1.65 to −0.687; *p* = 0.0001; *I* ^2^ = 74.01%) and BMI = 30–35 (*g* = −1.37; 95% CI, −1.86 to −0.877; *p* = 0.0001; *I* ^2^ = 77.35%).

**FIGURE 9:**
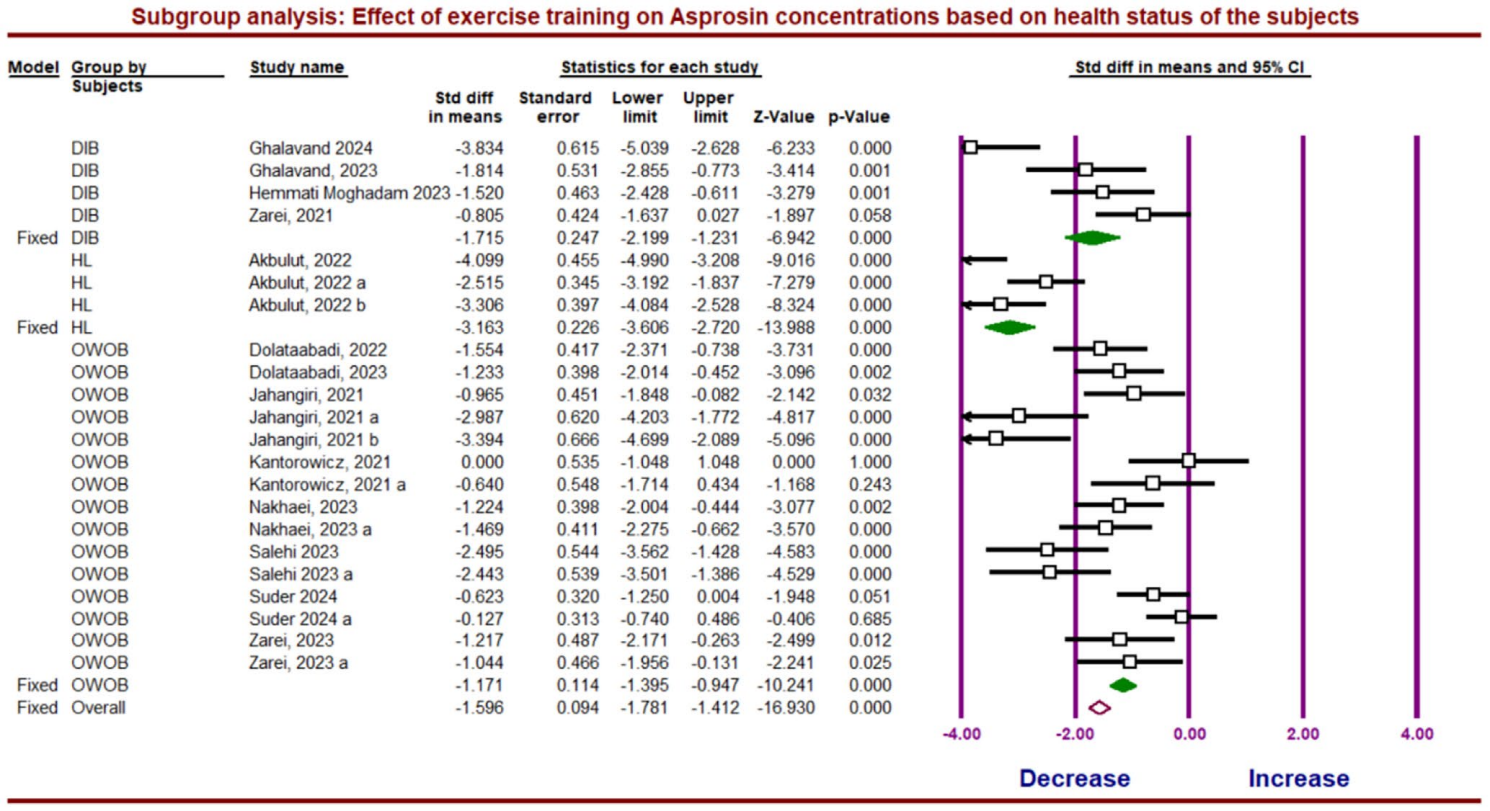
Forest plot summarizing subgroup analysis of asprosin protein response to exercise training based on health status of the participants. DIB, Diabetics; HL, Healthy subjects; OWOB, Overweight/obese subjects.

The subgroup analysis, based on the health status of the subjects, demonstrated significant differences in asprosin concentration between healthy, overweight/obese, and diabetic subjects (*Q*‐value = 12.647, df = 2, *p* = 0.002) (Figure 9). The within‐group changes demonstrated a significant reduction in asprosin levels due to ET in healthy participants (*g* = −3.16; 95% CI, −3.60 to −2.72; *p* = 0.0001; *I* ^2^ = 74.64%), overweight/obese (*g* = −1.13; 95% CI, −1.75 to −0.862; *p* = 0.0001; *I* ^2^ = 73.38%) and diabetic subjects (*g* = −1.17; 95% CI, −1.39 to −0.94).

## 4 DISCUSSION

The key finding from our meta‐analysis of 14 studies investigating the effect of ET is decreased asprosin that was unaffected by the type of training, sex, and duration of training. These findings indicate that long‐term training reduces asprosin regardless of whether the training is RT, although one study has shown that spinning exercises have a greater effect on reducing circulating asprosin than stationary cycling (Nakhaei et al., 2022). While no study has yet explored the mechanisms involved in reducing asprosin with ET, the primary source of asprosin production is considered to be fat tissue, meaning a reduction in fat mass with ET would decrease asprosin (Akbulut et al., 2022; Jahangiri et al., 2021; Nakhaei et al., 2022). Given the direct relationship between blood asprosin and glucose, HOMA‐IR, and fasting glycemia (Kantorowicz et al., 2021), and their association with asprosin reduction, it appears that ET‐induced metabolic adjustments such as reducing insulin resistance, blood sugar, and altering insulin and glucagon‐dependent pathways and changes in cAMP could be potential mechanisms that need further investigation.

Our meta‐analysis demonstrated significant reductions in circulating asprosin levels following exercise training (ET) in both overweight (BMI = 25–29.9; *g* = −1.53) and obese (BMI = 30–35; *g* = −1.37) individuals. While the effect sizes were large in both groups, the significant between‐subgroup heterogeneity (*Q* = 11.198, *p* = 0.004) suggests that BMI may modulate the response to ET, though the clinical magnitude of this difference appears modest. The observed reductions could be attributed to physiological adaptations induced by regular ET, including reductions in adipose tissue mass and improved metabolic function (Huang et al., 2022). Notably, individuals with obesity often exhibit elevated baseline asprosin levels due to metabolic dysfunction (Bengin et al., 2024; Mazur‐Bialy, 2023), which may make them more responsive to interventions targeting adipose‐derived hormones. Additionally, the interaction between asprosin and inflammatory markers suggests that ET‐induced reductions in asprosin may contribute to the mitigation of low‐grade inflammation, particularly in obesity (Guo et al., 2024). However, the similar effect sizes across BMI categories imply that the benefits of ET on asprosin are robust regardless of adiposity status. The slightly greater reduction in the overweight group may reflect differences in baseline metabolic health, study design, or measurement variability. The high heterogeneity (*I* ^2^ > 70%) within both subgroups underscores the need for further research to clarify the dose–response relationship between ET, adiposity, and asprosin regulation.

Mechanistically, ET enhances anti‐inflammatory myokine secretion, improves adipose tissue oxygenation, and promotes immune modulation (Guo et al., 2024; van Marken Lichtenbelt & Schrauwen, 2011), which may explain the pronounced effects in populations with elevated metabolic risk. Nevertheless, our findings suggest that ET effectively lowers asprosin across a broad BMI range, reinforcing its therapeutic potential for metabolic disorders.

We also found no effect of exercise duration on the magnitude of decline in asprosin that is in accord with earlier suggestions that a minimum period of 4 weeks of exercise is required to observe any effect (Kantorowicz et al., 2021). This requires further investigation. Moreover, there may be a threshold effect where the benefits of ET on asprosin are quickly achieved within the first weeks of ET.

Our study also found that the asprosin response to ET was not influenced by the participant's sex. While some individual studies (Ke et al., 2020; Long et al., 2019) report sex‐specific trends in asprosin response, our meta‐analysis found no significant difference in the magnitude of reduction between sexes (male: *g* = −1.78, 95% CI [−2.41, −0.16]; female: *g* = −1.57, 95% CI [−2.33, −0.81]). Also, it should be mentioned that the general trend is that both experience changes following ET (Ceylan et al., 2023; Wiecek et al., 2018). The similar effect sizes suggest that ET impact on asprosin may be mediated primarily by other factors (e.g., baseline adiposity, metabolic health, or exercise modality) rather than biological sex.

### 4.1 Limitations

This study is the first meta‐analysis to examine the impact of ET on asprosin. Given that this systematic review and meta‐analysis focused on individuals over the age of 18, it could be beneficial for future meta‐analyses to explore the impact of ET on asprosin in children and teenagers in which metabolic regulation differs (Chung & Rhie, 2022; Wittcopp & Conroy, 2016). The number of studies was limited, and the potential influence of unpublished, unidentified, and unretrieved studies on our results cannot be dismissed. To achieve a more accurate and definitive overall effect size, more studies need to be conducted and subsequently re‐evaluated.

## 5 CONCLUSION

Chronic ET for 8 weeks or longer results in reduced circulating asprosin in both men and women, which is enhanced in overweight and obese subjects. These findings suggest that ET is associated with a decrease in circulating asprosin, potentially benefiting metabolic health and preventing diseases exacerbated by obesity. Future research should explore the long‐term effects of ET on asprosin and its downstream metabolic pathways, as well as the potential for personalized exercise regimens based on individual asprosin levels and health status.

## AUTHOR CONTRIBUTIONS

M.R.R., M.E.S., and H.F. were responsible for the conceptualization, methodology, statistical analysis using CMA 3.0, investigation, visualization, and supervision of the study. M.R.R., M.E.S., H.F., and H.G. were involved in developing the methodology for systematic review and meta‐analysis, curating the data, and writing the initial draft of the manuscript. All authors have reviewed the manuscript and consent to its content.

## FUNDING INFORMATION

This study did not receive any specific grant from funding agencies in the public, commercial, or not‐for‐profit sectors.

## CONFLICT OF INTEREST STATEMENT

The authors declare that they have no competing interests.

## ETHICS APPROVAL

Not applicable.

## CONSENT FOR PUBLICATION

All authors declare their consent for the publication of this article.

## Supporting information


Appendix S1.

## Data Availability

As this is a meta‐analysis, all data included in this study are available in the cited references.
